# Enhancing Quality of Life: Key Factors from Long-Term Social Care Residents’ Perspectives

**DOI:** 10.3390/ijerph22020231

**Published:** 2025-02-06

**Authors:** Agrita Borovika, Martins Moors, Maksims Zolovs

**Affiliations:** 1 Department of Rehabilitation, Riga Stradins University, LV-1007 Riga, Latvia; 063131@rsu.edu.lv; 2 Riga State City Welfare Department, LV-1010 Riga, Latvia; maksims.zolovs@du.lv; 3 Institute of Life Sciences and Technology, Daugavpils University, LV-5401 Daugavpils, Latvia; 4 Statistics Unit, Riga Stradins University, LV-1007 Riga, Latvia

**Keywords:** social care system, Latvia, social care institution, long-term care, quality of life, self-assessment, clients

## Abstract

The purpose of this study was to investigate the most important factors shaping the quality of life in a social care institution from the clients’ perspective. This study examines factors beyond direct care that affect the quality of life for clients in social care institutions, focusing on Riga Municipality’s Social Care Center “Gailezers”. Structured interviews with 95 clients assessed six domains: autonomy, relationships, activities, environment, food, and care quality. This study emphasizes the need to align services with clients’ views to enhance their well-being. Based on the results, this study aimed to develop practical proposals for adjusting the content and organization of social care services to better align with the clients’ understanding of the factors affecting their quality of life in the institution.

## 1. Introduction

Societal aging is a global phenomenon, evident not only in Latvia but also in other countries worldwide, including those in Europe. For instance, South Korea, with the lowest fertility rates among OECD economies, is rapidly aging; by 2025, it is projected to become an ultra-aged society, where over 20% of the population will be 65 or older [1]. Aging has become a serious socio-demographic problem in Latvia after the restoration of national independence in the early 1990s. The primary driver of this phenomenon is a sustained period of extremely low fertility rates, insufficient to maintain population replacement. Latvia is one of the countries where the population is aging the fastest [2]. At the beginning of 2021, Latvia had 1,893,200 inhabitants, of which 393,698 were seniors aged 65 and over, making up 20.8% of the population. By contrast, 30 years ago in 1991, the proportion of senior citizens was nearly half as low at 11.8%, and in 2003, it had risen to 15.8%. Although Latvia’s total population is decreasing, the number of elderly people is rising [3]. As society ages, the number of people requiring social care services, both in the community and in social care institutions, is also increasing. For example, in Riga—the capital of Latvia—the number of long-term social care recipients was 2236 in 2023, 2196 in 2022, and 2045 in 2021 [4], indicating a steady rise in social care needs.

In Latvia, the Law on Social Services and Social Assistance stipulates that institutional social care is available only to individuals whose substantial care needs exceed the support provided by home care, day care centers, or other community-based services, with eligibility determined through a standardized, government-regulated evaluation tool uniformly applied nationwide. Therefore, only individuals with significant care needs are placed in social care institutions.

Many social care institutions in Latvia—built in the 1970s and 1980s as large facilities housing 100–300 clients—face challenges in meeting the modern standards of favoring family like environments, prompting funding plans from 2021 to 2027 to construct such facilities through the European Social Fund and Recovery and Resilience Mechanism support [5]; meanwhile, community-based services remain a slow-developing focus [6], leaving institutional care crucial and necessitating research on quality-of-life improvements.

Latvia’s regulations for long-term social care institutions [7] mandate round-the-clock supervision, self-care assistance, cognitive and movement activities, recreational and educational opportunities, and client provisions such as meals, hygiene supplies, and technical aids, with general facility requirements outlined, although they lack alignment with personalized views on quality of life.

To improve the quality of services in social care institutions, it is essential to understand clients’ opinions. This ensures that, given limited resources, investments are targeted and aligned with the specific needs and preferences of the clients.

### 1.1. Social Care Provision and Needs Assessment System in Latvia

The social services system in Latvia is governed by the Law on Social Services and Social types: social care services, social rehabilitation services, and social work services. Additionally, the LSSSA distinguishes between two modes of provision for social care and social rehabilitation services ([8], Section 22): (1) at the individual’s place of residence or nearby (also referred to as community-based or alternative social services, emphasizing their role as alternatives to institutional services); and (2) in long-term social care and rehabilitation institutions. Community-based services allow clients to remain in their usual residence while receiving support, such as home care provided directly at home or day care services offered nearby, with clients returning home at night and staff available only during limited hours; institutional social services, such as long-term care, rehabilitation centers, crisis centers, and shelters, require clients to change their place of residence and provide round-the-clock staff availability [9]. Social care services in institutions are associated with clients having greater needs for care and supervision.

In Latvia, social care services in institutions are provided only when clients’ care needs exceed what can be managed through home or day care services, as outlined in sections 1 and 18 of the LSSSA ([8], Section 28). This defines social care as measures addressing basic needs for individuals unable to perform self-care due to age or functional impairments, with the aim of maintaining their quality of life. According to the LSSSA and Cabinet of Ministers regulations [10], the municipal social service assesses residents’ needs and abilities using the unified methodology to determine appropriate social care services, with institutional care designated for individuals whose objectively assessed needs exceed the support offered by community-based services. For example, in the municipality of Riga, 35 h of home care per week is defined as the upper threshold; clients exceeding this amount are deemed eligible for placement in a social care institution [11]. This indicates that only clients with the highest care needs are granted the right to receive services in a social care institution.

According to Latvia’s social care legislation, maintaining quality of life in social care involves substituting the activities a person can no longer perform due to age or functional disorders while ensuring the support provided aligns with the goal of enabling independence, self-determination, and personal responsibility, offering help only for needs that cannot be met through personal effort, family, community, or technical aids. However, excessive support can undermine autonomy, and current regulations do not require social care services to address psychological needs, which are a critical aspect of quality of life. One of the aims of this study is to highlight the importance of factors beyond direct care in enhancing the quality of life for clients receiving social care services in an institution.

### 1.2. Factors Influencing Quality of Life in Social Care Institutions

The concept of quality of life is broad and encompasses all aspects of life but the scientific literature has yet to reach a consensus on its exact dimensions and variety. There is also the view that, at least in the Western world, most people are familiar with the term “quality of life” and have an intuitive understanding of what it entails [12].

Quality of life is understood as a combination of multiple domains, including housing, health, education, income, exposure to crime, leisure, culture, and access to green spaces. Additionally, the literature on quality of life distinguishes between objective and subjective aspects, recognizing the importance of individuals’ satisfaction with these and other domains. These characteristics likely facilitate the interaction between scientific knowledge, measurement tools (such as indicators), and specific policy goals and interventions [13]. However, it is clear that ”quality of life” means different things to different people and takes on various meanings depending on the context in which it is applied [12]. This indicates that quality-of-life research should encompass both subjective assessments from individuals and the context and environment in which they live.

Quality-of-life dimensions encompass physical well-being related to health, material well-being tied to economic stability and access to resources, interpersonal relationships involving social connections and support, personal development and self-determination reflecting potential realization and independent decision-making, as well as emotional well-being, social inclusion, and rights, all of which contribute to overall well-being [14]. It can be concluded that while the understanding of this concept varies among researchers and contexts, quality of life generally includes several essential components.

A study involving researchers from medicine, nursing, behavioral, and social sciences across 21 countries highlights well-being as a multifaceted concept ranging from a state of happiness to a subjective experience influenced by personal preferences, emphasizing in aged care the need for culturally valid measures that incorporate dimensions such as mobility, self-care, psychological and physical health, social relationships, environment, dignity, and control over daily living, thereby underscoring a multidimensional approach to evaluating quality of life and well-being in long-term care [15].

According to Linda S. Noelker, Ph.D., and Zev Harel, distinguishing between “quality of care” and “quality of life” in social care institutions simplifies measurement by enabling a clearer evaluation of care delivery effectiveness and clients’ life satisfaction, allowing for targeted improvements in health care, emotional well-being, or social support to enhance residents’ overall quality of life [16].

British researchers highlight that, although quality of care and quality of life are interconnected, they are not interchangeable as a person’s well-being can surpass the quality of care received or remain low despite excellent care, emphasizing the need to respect diverse cultural backgrounds and individual preferences—including personal and family definitions of a good quality of life—in social care institutions [17].

A Japanese research group, using the Donabedian model to categorize care quality into structure, process, and outcome, developed quality indicators identifying eight domains—maintaining dignity, minimizing symptoms, sustaining nutrition, maintaining continence, encouraging physical activity, promoting sleep, minimizing cognitive decline disabilities, and preserving family well-being—while emphasizing that client-reported life satisfaction and proxy-rated quality of life are essential but partial components of long-term-care evaluation in institutions [18]. This highlights the need to examine the interrelationships between different dimensions of quality.

In their book, *Care-related Quality of Life in Old Age: Concepts, Models, and Empirical Findings*, Marja Vaarama, Richard Pieper, and Andrew Sixsmith define the key criteria for quality of care as adequacy—ensuring that care aligns with individual needs and preferences; continuity—providing consistent and stable support; and professional competence—ensuring care workers deliver the highest standards and quality [19].

From the above, addressing the issue of quality of life and quality of care is complex as it fundamentally shapes how care quality is conceived, ensured, evaluated, and regulated in social care institutions. It is important to recognize that the clients’ understanding of quality of life is significantly impacted by these factors.

Jennifer L. Johs-Artisensi, a professor at the Department of Management and Marketing at the University of Wisconsin and an associate professor at Bellarmine University, along with Kevin E. Hansen, Chief of the Department of Health and Aging Services Management, offer a valuable perspective on addressing the interrelationship between quality of life and quality of social care in institutional settings. In their recently published book, “*Quality of Life and Well-Being for Residents in Long-Term Care Communities: Perspectives on Policies and Practices*” [20], Jennifer L. Johs-Artisensi and Kevin E. Hansen identify key factors that long-term social care recipients recognize as impacting their quality of life within social care institutions. The factors influencing the quality of life identified by the authors are as follows: (1) autonomy, respect, and sense of purpose; (2) relationships; (3) activities; (4) food and meals; (5) environment; and (6) quality of care. According to their classification, quality of care is just one of the factors contributing to the overall quality of life in a social care institution. This approach appears to bridge the gap between quality of life and quality of care by integrating both concepts within the context of long-term social care institutions.

Similar findings from research involving residents, families, and staff in care homes reveal that critical factors contributing to quality of life—such as security, belonging, continuity, purpose, achievement, and significance—apply to both clients and staff, as employees’ sense of safety, value, and belonging influences their relationship with clients, the quality of care they provide, and ultimately the residents’ self-assessed quality of life, which is closely linked to quality-of-care indicators [17,20]. Consequently, evaluating quality of life in a social care institution requires clients to complete self-assessments to capture their subjective experiences, priorities, and satisfaction with their living conditions and care.

One of the models of quality of life is the Patient-Preference Model, which differs from other models by explicitly incorporating weights that reflect the importance patients place on specific dimensions of their lives. This model involves comparing different states and dimensions to establish a ranking based on their value or the patients’ preferences for one state over another [12]. In assessing the quality of care, the person-centered model serves as a significant theoretical framework by defining care quality through the individual’s well-being, where quality of life—viewed from the client’s perspective—acts as a key indicator and plays a crucial role in the evaluation process [21]. Some authors argue that subjective methods are preferable for planning and policy as they capture personal feedback, highlight dissatisfaction, and support a bottom-up approach to addressing community-based issues and improving social care [13].

The aim of this study is to convert the feedback obtained from clients into actionable proposals for enhancing the services provided by social care institutions. This research focuses on the Riga Municipality Social Care Center “Gailezers” and aims to extend the findings to similar social care institutions.

The authors of this study, who conducted self-assessments with long-term care clients, acknowledge that all participants required assistance, often of a more intensive nature, to maintain orientation, focus, and comprehension to complete the questionnaire [22]. Therefore, the structured interview method was employed in this study. With this approach, questions were asked and, if needed, explained by employees of the social care institution who were not directly involved in performing care tasks.

## 2. Materials and Methods

The research base was the Riga Social Care Center “Gailezers” (hereinafter the Center), which provides long-term social care services in a social care institution.

A total of 328 clients were receiving services at the Center at the time of this study. Due to limitations in the chosen research strategy and methods, not all clients’ opinions could be gathered as the assessment required certain cognitive and physical abilities, including the ability to articulate feelings and opinions. During the research, 95 clients were interviewed using the structured interview method. Clients with dementia or signs of dementia, those with severe functional impairments who were sedentary, clients with hearing and speech impairments, those in hospital, clients who were absent, and those who refused to participate were not included in the interviews.

A structured interview was conducted using a checklist (Supplementary Materials) of interview and observation questions adapted from Jennifer L. Johs-Artisensi and Kevin E. Hansen [20]. This checklist was specifically designed for assessing six key domains of quality of life for clients residing in a long-term care facility. The interview questions were organized into six groups based on the factors determining the quality of life in a social care institution, as identified by the authors: (1) autonomy, respect, and sense of purpose; (2) relationships; (3) activities; (4) environment; (5) food and meals; and (6) quality of care.

The interviews were conducted between 22 July and 2 August 2024 by 10 employees of the social care center whose regular work responsibilities did not involve direct interaction with the clients. Structured interviews, “which closely resemble quantitative surveys in their characteristics” ([23], p. 296) involve asking all participants a standardized set of questions in a consistent order. This approach minimizes interviewer bias, ensures data comparability across respondents, and yields higher reliability, validity, and predictive accuracy compared to unstructured formats. The validation of the structured interview questions involved a rigorous translation and back-translation process to ensure cross-cultural equivalence, starting with independent translations from English to Latvian and resolving discrepancies through consensus. The reconciled Latvian version was then back-translated into English by different translators, and any differences were addressed collaboratively to ensure conceptual equivalence. Finally, the Latvian version of the structured interview questionnaire was pilot-tested with five participants from the target population, leading to minor revisions based on feedback to enhance the clarity and validity.

Structured interviews, as a type of survey research [24], enable researchers to capture subjective opinions in a standardized format while producing data suitable for quantitative analysis. This method was chosen due to the significant care needs of the clients, which rendered self-administered questionnaires impractical. By allowing interviewers to orally administer standardized questions, structured interviews ensure inclusivity and facilitate participation regardless of literacy or physical limitations. Furthermore, they achieve higher response rates, minimize interviewer bias, and provide consistent, objective, and comparable data, enabling the aggregation and statistical analysis of participants’ responses. This study was conducted in accordance with the Declaration of Helsinki and approved by the Research Ethics Committee of Riga Stradins University, Latvia. Informed consent was obtained from all subjects involved in this study.

### Statistical Data Analysis

To assess quality-of-life factors, a Likert-type scale was employed in the questionnaire. Respondents indicated their level of agreement with each statement on a five-point Likert scale ranging from 1 (strongly disagree) to 5 (strongly agree). Composite scores were calculated by summing item responses within each factor, as well as all factors together (overall composite score). This resulting sum score was utilized as the dependent variable in subsequent statistical analyses to test the statistical hypothesis where the independent variables were age, time spent at the Center, language of daily interactions, education, relatives, and the number of people in a room.

The data distribution was assessed by inspection of the normal Q-Q plot and with the Shapiro–Wilk test. For normally distributed data, mean values and standard deviations were reported. In contrast, the median and interquartile range (IQR) were used to summarize non-normally distributed data. Nominal data were described by frequencies and percentages.

The Mann–Whitney U test was used to compare composite scores between the language of daily interactions and relatives. The Kruskal–Wallis H test was used to compare composite scores between the number of people in the room and education levels. To test the association between age, time spent at the Center, and the composite scores of life factors, Spearman’s rank correlation was used. Correlation coefficients were interpreted according to Cohen’s (1988) guidelines [25].

A linear regression analysis was conducted to test whether age, time spent at the Center, language of daily interactions, education, relatives, and the number of people in the room were related to overall composite scores. To build the regression model, stepwise forward and backward regression methods were used. To select the best model, the Akaike Information Criterion (AIC) and adjusted R^2^ were used.

Statistical data analyses were performed with Jamovi (v.2.3). The results were considered statistically significant when the *p* value was <0.05.

## 3. Results

The analysis revealed several significant findings that shed light on the assessment of factors influencing the quality of life in a social care institution according to the clients’ opinions. Table 1 provides the parameters of the clients that were analyzed.

Approximately half of the respondents were Latvian speakers, while the other half primarily used Russian in their daily conversations. The average age of the interviewees was 75.5 years. Two-thirds of the clients had either secondary or technical secondary education, two-thirds lived in a room alone, while 26% of the clients shared their living space with a roommate.

The results of Cronbach’s alpha (overall = 0.95) indicate a strong internal consistency among the questions (Table 2). This suggests that the modified interview questionnaire is effectively designed for use alongside the Likert scale, ensuring reliable measurement of client responses. However, when examining the assessment of different quality-of-life factors individually, Cronbach’s alpha for the “environment” factor falls below the threshold of 0.7. The distribution of the scores presented in Table 2 is depicted as a bar chart in the Supplementary Materials.

The recalculated Likert scale ratings reveal that clients rate the “relationship” factor the highest, while the “environment” factor receives the lowest rating in the social care institution where they reside.

The customer ratings do not statistically significantly differ between those who have or do not have relatives (*p* > 0.05), nor between different levels of education (*p* > 0.05). No difference was observed in the length of stay in the Center (*p* > 0.05). However, a statistically significant difference (*p* < 0.05) was found in the evaluations based on everyday language for criteria such as autonomy, respect, and sense of purpose, relationships, and quality of care (see Table 3). Russian speakers rated these criteria higher than Latvian speakers. Judging by the effect size coefficient, the differences in the indicators for autonomy, respect, and sense of purpose, relationships, and quality of care are moderately large, with the coefficient ranging between 0.2 and 0.5. However, for the quality-of-life factors related to activities, environment, and food and meals, there are no significant differences between the evaluations of Latvian-speaking and Russian-speaking clients (*p* > 0.05).

In general, the overall difference indicates that there is a significant variation in quality-of-life assessments based on the client’s language (*p* = 0.017, effect size = 0.5). On average, Latvian-speaking clients rate their overall quality of life lower (overall score = 170 ± 27.6) compared to those who use Russian (overall score = 183 ± 23.6) in their daily lives. Latvian speakers also exhibit a higher standard deviation in their assessments compared to Russian speakers. This indicates that Latvian speakers have more varied opinions with a broader range of responses, whereas Russian speakers’ opinions are more consistent and less extreme.

Table 4 shows the data on the differences in ratings based on whether a client lives alone or shares a room with others. The *p*-value is below 0.05 for the environment factor, autonomy, respect, and sense of purpose, and the overall assessment. Clients living with roommates tend to rate the environment factor, autonomy, respect, and sense of purpose, and the overall quality of life lower than those living alone. This suggests that increased room occupancy is associated with more critical assessments of the environment, autonomy, respect, and sense of purpose, and overall quality of life in the social care institution.

The final linear regression model was significant F(2,92) = 7.86, *p* < 0.001, adj R^2^ = 0.13, indicating approximately 13% of the variance in overall composite score is explained by the language of daily interactions and the number of people in the room. Language significantly predicted the overall composite score (β = 12.6, t = 2.50, *p* = 0.014). Russian speakers scored, on average, 12.6 points higher on the overall composite score than Latvian speakers (95% CI [2.6-22.7]). The factor of other people in the room significantly predicted the overall composite score (β = 15.7, t = 3.05, *p* = 0.003). People living alone in a room scored, on average, 15.7 points higher on the overall composite score than those who lived in a room with roommates (95% CI [5.5–25.9]).

**Figure 1:**
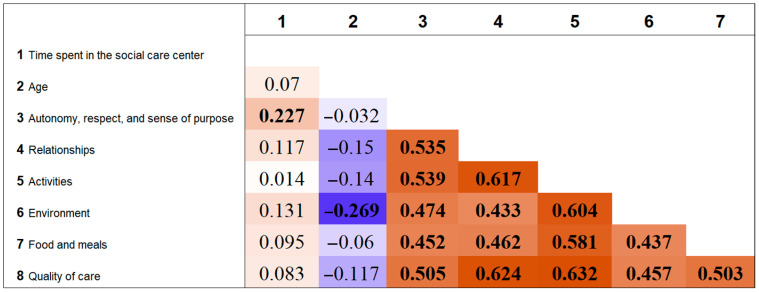
Correlation matrix of quality-of-life factors and clients’ age and time spent at the social care institution. Bold numbers indicate statistically significant correlations (*p* < 0.05). Positive correlations are shown in brown, while negative correlations are shown in blue. Color intensity reflects the correlation’s magnitude: darker shades indicate stronger correlations, and lighter shades indicate weaker correlations.

The analysis shows that the more time a client spends at the Center, the higher the rating given to the autonomy factor (r_s_ = 0.227). Conversely, age correlates negatively with the environment factor, meaning that older clients tend to rate the environment of the Center lower (r_s_ = −0.270), indicating that the environment of the Center may be less suitable for the oldest clients (Figure 1).

The strongest correlations were found between SS2—relationships and SS3—activities (r_s_ = 0.617); SS2—relationships and SS6—quality of care (r_s_ = 0.624); SS3—activities and SS4—environment (r_s_ = 0.604); and SS3—activities and SS6—quality of care (r_s_ = 0.632).

Clients and employees of the Center were asked to rank the six key factors of quality of life, as identified by Jennifer L. Johs-Artisensi and Kevin E. Hansen [19], in order of importance. The most crucial factor was assigned the number “1”, while the least crucial was assigned the number “6”. The results of this ranking are presented in Table 5.

The results indicate that the ratings given by clients and employees are nearly identical. The primary distinction is that clients prioritize autonomy, respect, and sense of purpose as the most crucial factor affecting their quality of life in the social care institution, with quality of care as the second most important factor. In contrast, employees rank quality of care as the top priority, followed by autonomy, respect, and sense of purpose. Both clients and employees rank relationships as third, food and meals as fourth, and environment as fifth, with activities being considered of relatively lower priority. Consequently, autonomy and quality of care emerge as the most crucial factors for maintaining quality of life in a social care institution.

In addition to the overall statistical results, the researchers focused on identifying the specific individual questions from the structured interview that received the most critical evaluations from the clients. The purpose was to identify precisely which measures should be taken to address the most critically evaluated specific issues related to quality-of-life factors.

The lowest customer rating was given to question 4.4: “how often can you leave the facility on Center resident outings (excursions, etc.)?” This question was part of the environment factor. The average rating given by customers was “3”, indicating that outings occur “sometimes (about 3 times a year)”. The interquartile range was from 1 to 5, reflecting considerable variability in the responses. A similar situation was observed in question 5.2, which asked, “Do you feel that you have some choice about what food to eat at each meal?” This question, part of the food and meals factor, received the second-lowest rating, indicating that clients felt they had limited choice regarding their meals. The third-lowest rating was given to question 6.2, “Do you think carers like their jobs?” which was part of the quality of care factor. The lower rating for question 6.2 suggests that clients may perceive a lack of enthusiasm or job satisfaction among carers, which could impact their overall perception of the quality of care provided. This highlights the need for further investigation into staff morale and its effects on client satisfaction.

## 4. Discussion

Several key insights have emerged from the analysis of the clients’ feedback, underscoring areas for improvement within the institution and providing a path for enhancing the overall quality of care.

The findings that clients rated the “relationship” factor highest and the “environment” factor lowest at the social care institution suggest a clear distinction between the perceived importance of social connections and physical surroundings in shaping overall well-being. This aligns with the conclusions of other researchers who emphasize that the quality of the interaction between clients and care workers is crucial as positive relationships foster trust and satisfaction, enhancing clients’ well-being and indirectly improving care workers’ effectiveness [19].

Additionally, data from the evaluation of the quality-of-life factor the “environment” show differences in the ratings based on whether a client lives alone or shares a room with others. Other researchers highlight that a living environment tailored to subjective needs—such as the inclusion of personal items and attention to social–emotional, cultural, and organizational contexts—can significantly enhance a client’s quality of life [19], while participants in our research placed greater emphasis on the number of people sharing a room as a critical factor in their responses. Clients living with roommates tend to rate the environment factor, autonomy, respect, and sense of purpose, and overall quality of life lower than those living alone. This suggests that increased room occupancy is associated with more critical assessments of the environment, autonomy, respect, and sense of purpose, and overall quality of life at the social care institution.

This is consistent with the results of another study conducted in Latvia in 2016-researchers from the University of Latvia conducted a study in which the opinion of experts was ascertained through semi-structured interviews. During the interview, the social rehabilitator pointed out that the main challenge in long-term social care institutions is the ability to provide the elderly with single rooms in long-term institutions. Another issue that was highlighted was the size of the rooms, which should be bigger giving more space for its residents [26]. This indicates that the environment factor is one of those that should be given priority attention when improving the service of a social care institution.

The results also show a negative correlation between age and the environment factor, indicating that older clients tend to rate the Center’s environment lower, suggesting it may be less suitable for them. As age increases, clients’ perceptions of the environment become more negative, implying that older clients may find the surroundings less accommodating or comfortable. This suggests that environmental factors play an increasingly significant role in the quality of life for older clients in social care institutions. Therefore, the appropriateness, comfort, aesthetics, and accessibility of the environment should be specifically assessed and tailored to meet the needs of older clients.

The client ratings show no statistically significant differences between those with or without relatives. This result was unexpected, especially regarding the presence of relatives, as it is commonly assumed by social care staff that clients with family connections are more likely to be critical of living conditions in social care institutions. However, the survey data do not support this assumption.

It is recommended that the Center’s administration investigate the factors contributing to the more critical evaluations from Latvian-speaking clients and explore strategies to reduce room occupancy. A statistically significant difference was identified in the assessments of autonomy, respect, and sense of purpose, relationships, and quality of care based on clients’ everyday language, with Russian-speaking clients consistently rating these factors higher than Latvian speakers. This disparity suggests the need to examine potential underlying causes. Latvian speakers reported a lower overall quality of life, highlighting the importance of targeted interventions to address their specific concerns. Further research is necessary to explore whether these differences stem from cultural, psychological, staff communication practices, or other factors to better understand and address the issues.

Discussion about what should be implemented to improve the quality of life of clients at social care institutions that cannot renovate, refurbish, or rearrange the environment to be modern, family like, and correspond to an up-to-date understanding of a decent care environment should take into account the correlations between different quality-of-life factors according to the clients’ opinion. If, as noted, the environment is rated most critically by clients, then—based on the correlations presented in the Section 3—it can be assumed that expanding the range of activities could positively influence clients’ perceptions of the Center’s environment or help mitigate its shortcomings. Similarly, enhancing relationships could encourage greater participation in activities and improve clients’ perceptions of the quality of care. The results of other studies indicate that individuals who increased their participation in physical activity reported better mental health perceptions, especially in aspects of social and emotional functioning, compared to those who reduced their activity levels [27]. However, while physical activity is generally beneficial for health, some studies indicate that it contributes less to the quality of life for institutionalized elderly individuals compared to their community-dwelling counterparts [28]. These findings suggest that social care institution administrations should carefully investigate the preferences of elderly residents to better align activities with their interests.

Such insights provide a valuable direction for improving the content of the social care institution. This approach is particularly crucial in post-Soviet buildings, where physical environment improvements are often limited; thus, compensating for these limitations through content and relational enhancements becomes a viable strategy. It can also be inferred that improving clients’ perception of the quality of care can be achieved by enhancing the quality of relationships, both among clients and between clients and caregivers. Research by Vieira and colleagues suggests that a supportive work environment fostering positive psychosocial factors—such as influence, development opportunities, meaningful work, commitment, role clarity, predictability, and effective leadership—enables well-qualified staff to significantly enhance the quality of care provided to residents [21]. These findings suggest that enhancing the working conditions of care workers also contributes to improving the quality of life for clients in social care institutions. Other researchers suggest emphasizing the concept of “accompaniment”, positing that supporting older adults, technical staff, and political sectors can create a beneficial chain reaction, enhancing service quality and promoting person-centered care through infrastructure renewal and the development of multifunctional, community-inclusive spaces that foster well-being and inclusion [29]. This provides the Center’s administration with evidence-based guidance on where to focus their efforts for the most effective solutions. Such findings are particularly valuable in the context of limited resources.

Some client responses highlight issues that require immediate attention in the social care institution’s service offerings, as indicated by the questions that received the most critical feedback. Clients’ most critical comments suggested that their quality of life would improve with more opportunities to go outside the institution, even minor influence over meal content, and greater enthusiasm and engagement from caregivers in their work. The issues related to food and their importance are highlighted in other research as well, which suggests that, among various factors influencing an individual’s quality of life, food-related concerns are often perceived as less favorable or more problematic [30], emphasizing the significance of the clients’ ability to influence meal content and processes in shaping their quality of life in social care institutions.

The attitudes of care staff and potential improvements are reflected in the assessments of clients and employees regarding the priority of various quality-of-life factors within the social care institution. Although the difference in opinions between clients and employees is not essential—clients prioritize autonomy, respect, and a sense of purpose as the most crucial factors affecting their quality of life, with quality of care ranked second—employees rank quality of care as their top priority, followed by autonomy, respect, and a sense of purpose. This suggests that, in situations where employees must choose between ensuring a client’s autonomy and providing care, they may prioritize care activities over respecting the client’s autonomy. It also underscores the significance of social policy in transitioning toward a service model that resembles a family environment, where small group settings and a focus on individualized care play crucial roles. It is evident that the Center’s culture and the perspectives of both clients and employees are relatively aligned concerning what is most critical for ensuring quality of life in a social care institution. This consensus indicates that the organizational environment and culture support collective improvement as there is substantial agreement between clients and care workers on the priority and relative importance of various quality-of-life factors within the institution. However, this also suggests that employees should receive training on the importance of autonomy, respect, and sense of purpose, and learn how to enhance clients’ opportunities to exercise these aspects.

Although the sample size was limited to 95 participants—less than one-third of the total resident population—and certain groups were excluded due to the necessity of cognitive abilities to express opinions on quality-of-life factors in the social care institution, this study still identifies key factors that influence well-being from the clients’ perspective. Despite these exclusions, the research highlights critical elements shaping clients’ experiences and perceptions of their quality of life, offering valuable insights into enhancing care services within the institution. The exclusion of individuals with dementia, severe functional impairments, and other challenges may have affected the generalizability of the results to the broader population of clients. Additionally, the composite factor of “autonomy, respect, and sense of purpose” needs further examination as these concepts are distinct and not directly related. Despite these limitations, this research provides meaningful directions for improving social care services by better aligning them with clients’ perceptions, offering a foundation for future studies to address these complexities and refine service delivery.

## 5. Conclusions

The findings of this study underscore the critical importance of relationships in determining the quality of life for clients in social care institutions as this factor consistently received the highest ratings, while the environment was rated lowest. Room-sharing emerged as a significant concern, with clients living alone reporting more positive evaluations of autonomy, respect, and overall quality of life, suggesting that privacy and personal space are essential for well-being. To address these concerns, social care institutions should consider strategies to reduce room occupancy and improve shared living conditions while prioritizing environmental adaptations that better cater to the needs of older clients.

This research also highlights notable disparities in quality-of-life assessments among client groups, particularly between Latvian-speaking and Russian-speaking residents, with the former reporting lower satisfaction across various dimensions. These findings suggest an urgent need for targeted interventions to address cultural, psychological, and communication barriers, as well as a comprehensive review of factors influencing these critical evaluations. Additionally, the lack of significant differences in assessments based on relationships with relatives or educational backgrounds challenges assumptions about these factors, emphasizing the importance of universally improving care standards.

Finally, enhancing relationships between clients and caregivers and expanding activity options could mitigate environmental shortcomings, particularly in facilities with limited potential for physical upgrades. Incorporating client feedback into service improvements, such as increasing outdoor activities and fostering caregiver engagement, is essential for creating a supportive and responsive care environment. Furthermore, improving working conditions and fostering a positive psychosocial environment for staff could significantly enhance the quality of care provided, leading to better client outcomes and a more holistic, client-centered approach to social care.

## Figures and Tables

**Table 1:** Characteristics of study group.

Parameters	Values
Age, mean (SD)	75.5 (10.3)
Time spent at the SCC in years, median (Q1–Q2)	3 (1.4–8.0)
Language of daily interactions, n (%)	
Latvian	48 (50.5)
Russian	47 (49.5)
Education level, n (%)	
Lower primary (grades 1–4)	7 (7)
Elementary (grades 1–9)	12 (13)
Secondary (grades 10–12)	29 (30)
Technical College	32 (33)
University	16 (17)
Relatives, n (%)	
Yes	69 (72)
No	27 (28)
Number of people in the room, n (%)	
1	56 (64)
2	23 (26)
4	8 (10)

Note: SSC—social care center; SD—standard deviation; Q1–Q3—the first and third quartiles.

**Table 2:** Descriptive statistics of quality-of-life factors in social care institution and results of reliability analysis.

Life Factors	Cronbach’s Alpha	Assessment (Likert Scale)	Composite Score
		Median (Q1–Q3)	Median (Q1–Q3)
Autonomy, respect, and sense of purpose	0.80	4 (4–5)	41 (38–46)
Relationships	0.80	5 (3–5)	26 (21–29)
Activities	0.78	5 (3–5)	20.5 (18–24)
Environment	0.60	5 (3–5)	16 (14–18)
Food and meals	0.81	4 (3–5)	27 (23–33)
Quality of care	0.88	4 (3–5)	49 (42–55)
**Overall**	0.95		180 (160–195)

**Table 3:** The comparison of composite scores between language of daily interactions.

Life Factors	Latvian	Russian	*p*	Effect Size
	Median (Q1–Q3)	Median (Q1–Q3)		
Autonomy, respect, and sense of purpose	41 (38–45)	42 (38–47.5)	0.119	NA
Relationships	26 (19–29)	26 (23–30)	0.039	0.25
Activities	20 (17–23)	21 (18.5–24)	0.389	NA
Environment	15.5 (14–17.3)	17 (14.5–19)	0.157	NA
Food and meals	27 (23–31)	28 (23.5–34)	0.147	NA
Quality of care	45.5 (40–54)	51 (45–58)	0.014	0.29
**Overall, mean (SD)**	170 (27.6)	183 (23.6)	0.017	0.50

Note: NA–not applicable.

**Table 4:** The comparison of composite scores between number of people in the room.

Life Factors	1 Person Per Room	2 People Per Room	4 People Per Room	*p*	ε^2^
	Median (Q1–Q3)	Median (Q1–Q3)	Median (Q1–Q3)		
Autonomy, respect, and sense of purpose	44 (40–46)	39 (32–41.5)	38.5 (38–41)	0.002	0.14
Relationships	28 (23–29)	23 (21–29)	24.5 (21–26)	0.062	NA
Activities	20.5 (18–24)	21 (18–24)	21 (16–22)	0.690	NA
Environment	17 (15–18)	16 (13–20)	14 (7.5–15)	0.007	0.11
Food and meals	27.5 (24–33)	27 (23–33)	26 (25–26)	0.680	NA
Quality of care	52 (45–56)	51 (41.5–54)	43.5 (42–46)	0.106	NA
**Overall**	185 (167–199)	170 (152–200)	169 (153–174)	0.023	0.08

Note: NA–not applicable; ε^2^–effect size.

**Table 5:** Significance of factors.

Quality-of-Life Factors	Clients	Workers
Autonomy, respect, and sense of purpose	276 (1st place)	217 (2nd place)
Relationships	319 (3rd place)	259 (3rd place)
Activities	428 (6th place)	482 (6th place)
Environment	330 (5th place)	389 (5th place)
Food and meals	329 (4th place)	386 (4th place)
Quality of care	313 (2nd place)	208 (1st place)

## Data Availability

The raw data supporting the conclusions of this article will be made available by the authors upon request.

## Supplementary Materials

The following supporting information can be downloaded at: https://www.mdpi.com/article/10.3390/ijerph22020231/s1, Figure S1: Distribution of quality-of-life scores depicted as bar chart figures; File S1: Quality of life questionnaire.
