# Supplementary material for: Enhancing Quality of Life: Key Factors from Long-Term Social Care Residents’ Perspectives
Source: Int J Environ Res Public Health. 2025 Feb 6;22(2):231. doi: 10.3390/ijerph22020231 (PMC11855746; doi:10.3390/ijerph22020231)
Supplement: Supplementary file 1 [file ijerph-22-00231-s001.zip › ijerph-3300022-Quality of life questionnaire.pdf]

Dati par respondentu:

Vecums:_______________________________

Sarunvaloda: __________________________

Izglītība: _____________________________

Cik ilgi dzīvo Centrā:___________________

Vai ir tuvinieki: _______________________

Vai dzīvo viens istabiņā: ______________

Jautājumu saraksts sociālās aprūpes pakalpojuma klientu intervijai, novērtējot Dzīves kvalitāti veidojošos faktorus ilgtermiņa sociālās aprūpes institūcijā

Atbildes tiek grupētas, izmantojot Likerta skalu. Likerta skala ir vērtēšanas skalas veids, ko izmanto, lai novērtētu attieksmi vai viedokļus, piedāvājot dažādas atbilžu iespējas no vienas galējības uz otru. To parasti veido virkne apgalvojumu, kuriem respondenti norāda savu piekrišanas vai nepiekrišanas līmeni vairāku punktu skalā. Likerta skala izmanto intervālu atbilžu formātu, parasti no 1 līdz 5. Šajā skalā mazākais un lielākais skaitlis apzīmē divas pretējas galējības, piemēram, "pilnībā piekrītu" un "pilnībā nepiekrītu". Tā tiek izmantota, lai mērītu respondentu attieksmes pret dažādiem jautājumiem vai izteikumiem.

1.faktors Autonomija, cieņa un mērķa izjūta

1.1. Vai jums patīk šeit dzīvot?

Ļoti nepatīk	Drīzāk nepatīk	Neitrāli	Drīzāk patīk	Ļoti patīk

1.2. Vai jums šķiet, ka jums šeit ir mērķa izjūta, vai centrā dzīvošana ir saturīga un pilnvērtīga?

Nepiekrītu	Drīzāk nepiekrītu	Neitrāli (ne piekrītu, ne nepiekrītu)	Drīzāk piekrītu	Piekrītu

1.3. Vai jūs jūtaties piederīgs mūsu sociālās aprūpes centra iemītnieku grupai

Nepiekrītu	Drīzāk nepiekrītu	Neitrāli (ne piekrītu, ne nepiekrītu)	Drīzāk piekrītu	Piekrītu

1.4. Vai jūtat, ka aprūpētāji ciena jūsu privātumu?

Nepiekrītu	Drīzāk nepiekrītu	Neitrāli (ne piekrītu, ne nepiekrītu)	Drīzāk piekrītu	Piekrītu

1.5. Vai jums šķiet, ka darbinieki ir veltījuši laiku, lai iepazītu jūs kā personu?

Nepiekrītu	Drīzāk nepiekrītu	Neitrāli (ne piekrītu, ne nepiekrītu)	Drīzāk piekrītu	Piekrītu

2. faktors - Attiecības

2.1. Vai jums liekas, ka **daži** no jūsu darbiniekiem (aprūpētājiem, soc. Apr. darbinieki utt) ir jums kā draugi vai tuvinieki (ģimenes locekļi), vai jūs domājat par dažiem saviem aprūpētājiem kā par draugiem vai ģimeni (tuviniekiem)?

Nepiekrītu	Drīzāk nepiekrītu	Neitrāli (ne piekrītu, ne nepiekrītu)	Drīzāk piekrītu	Piekrītu

2.2. Vai aprūpētāji ir labi klausītāji?

Nepiekrītu	Drīzāk nepiekrītu	Neitrāli (ne piekrītu, ne nepiekrītu)	Drīzāk piekrītu	Piekrītu

2.3. Vai viņi runā ar jums, kamēr viņi jums palīdz?

Nepiekrītu	Drīzāk nepiekrītu	Neitrāli (ne piekrītu, ne nepiekrītu)	Drīzāk piekrītu	Piekrītu

2.4. Vai darbinieki klauvē, pirms viņi ienāk jūsu istabā?

Nepiekrītu	Drīzāk nepiekrītu	Neitrāli (ne piekrītu, ne nepiekrītu)	Drīzāk piekrītu	Piekrītu

2.5. Vai viņi pēc iespējas saglabā jūsu privātumu, pildot aprūpes uzdevumus?

Nepiekrītu	Drīzāk nepiekrītu	Neitrāli (ne piekrītu, ne nepiekrītu)	Drīzāk piekrītu	Piekrītu

2.6. Vai aprūpētāji jūs sauc tādā vārdā (veidā), kā jūs vēlaties?

Nepiekrītu	Drīzāk nepiekrītu	Neitrāli (ne piekrītu, ne nepiekrītu)	Drīzāk piekrītu	Piekrītu

3. faktors - Aktivitātes

3.1. Vai jums šķiet, ka šeit ir daudz dažādu aktivitāšu, kurās jūs varat piedalīties?

Nepiekrītu	Drīzāk nepiekrītu	Neitrāli (ne piekrītu, ne nepiekrītu)	Drīzāk piekrītu	Piekrītu

3.2. Vai jums ir izvēle piedalīties aktivitātēs, kas jums patīk?

Nepiekrītu	Drīzāk nepiekrītu	Neitrāli (ne piekrītu, ne nepiekrītu)	Drīzāk piekrītu	Piekrītu

3.3. Vai jums ir iespēja darīt kādas aktivitātes vienatnē nevis grupā, ja to vēlaties? Vai Centrs piedāvā Jums ko individuāli, ko Jūs vēlaties (dators, bibliotēka, ēst gatavošana)?

Nepiekrītu	Drīzāk nepiekrītu	Neitrāli (ne piekrītu, ne nepiekrītu)	Drīzāk piekrītu	Piekrītu

3.4. Vai esat apmierināts ar aprūpes centra darbiniekiem, kā viņi uzklausa jūsu idejas par iespējamajām vai esošajām aktivitātēm?

Nepiekrītu	Drīzāk nepiekrītu	Neitrāli (ne piekrītu, ne nepiekrītu)	Drīzāk piekrītu	Piekrītu

3.5. Vai jūtat, ka jums ir daudz dažādu aktivitāšu, kurās piedalīties pat izolācijas vai karantīnas periodos?

Nepiekrītu	Drīzāk nepiekrītu	Neitrāli (ne piekrītu, ne nepiekrītu)	Drīzāk piekrītu	Piekrītu

4. faktors - Vide

4.1. Vai jūs jūtaties ērti šeit (vai jums ir komfortabli apstākļi)?

Nepiekrītu	Drīzāk nepiekrītu	Neitrāli (ne piekrītu, ne nepiekrītu)	Drīzāk piekrītu	Piekrītu

4.2. Vai jūs varat saņemt atbalstu, lai izmantotu pieejamās telpas tā, kā vēlaties (piemēram, frizētava, veikals, dārza telpas, bibliotēka)?

Nepiekrītu	Drīzāk nepiekrītu	Neitrāli (ne piekrītu, ne nepiekrītu)	Drīzāk piekrītu	Piekrītu

4.3. Cik bieži jums ir iespēja iziet ārā (ārpus SAC)?

Ļoti reti (apmēram 1 reizi mēnesī vai retāk)	Reti (apmēram vienu reizi divās nedēļās)	Dažreiz (apmēram reizi nedēļā)	Bieži (vairākas reizes nedēļā)	Ļoti bieži (reizi dienā vai biežāk)

4.4. Cik bieži jūs varat atstāt iestādi SAC iemītnieku izbraukumā (ekskursija un tml.)?

Ļoti reti (apmēram 1 reizi gadā vai retāk)	Reti (apmēram divas reizes gadā)	Dažreiz (apmēram trīs reizes gadā)	Bieži (četras reizes gadā)	Ļoti bieži (reizi mēnesī vai biežāk)

5. faktors - Pārtika

5.1. Kā jūs vērtētu ēdiena kvalitāti aprūpes centrā?

Pilnībā neapmierina	Drīzāk neapmierina	Neitrāli (reizēm apmierina, reizēm neapmierina)	Drīzāk apmierina	Pilnībā apmierina

5.2. Vai jūtat, ka jums ir kāda (kaut neliela) izvēle, kādu ēdienu ēst katrā ēdienreizē?

Nepiekrītu	Drīzāk nepiekrītu	Neitrāli (ne piekrītu, ne nepiekrītu)	Drīzāk piekrītu	Piekrītu

5.3. Vai jūs domājat, ka šeit pasniegtajās maltītēs ir pietiekami daudz dažādības (daudzveidīgas)?

Nepiekrītu	Drīzāk nepiekrītu	Neitrāli (ne piekrītu, ne nepiekrītu)	Drīzāk piekrītu	Piekrītu

5.4. Kā jūs vērtējat ēdienreižu porcijas lielumu? Vai ēdiena ir pietiekami daudz, lai paēstu?

Pilnībā neapmierina	Drīzāk neapmierina	Neitrāli (reizēm apmierina, reizēm neapmierina)	Drīzāk apmierina	Pilnībā apmierina

5.5. Vai starp ēdienreizēm ir pieejamas uzkodas?

Nepiekrītu	Drīzāk nepiekrītu	Neitrāli (ne piekrītu, ne nepiekrītu)	Drīzāk piekrītu	Piekrītu

5.6. Kā jūs vērtētu (ēdināšanas organizācijas un apstākļu – iekārtojums, dekorācijas, apgaismojums, mūzika un skaņas, apkalpošana u.c.) atmosfēru ēdienreižu laikā , maltīti ieturot ēdamzālē vai istabiņā?

Pilnībā neapmierina	Drīzāk neapmierina	Neitrāli (reizēm apmierina, reizēm neapmierina)	Drīzāk apmierina	Pilnībā apmierina

5.7. Vai jums patīk jūsu galda biedri un spēja socializēties ēdienreižu laikā?

Pilnībā neapmierina	Drīzāk neapmierina	Neitrāli (reizēm apmierina, reizēm neapmierina)	Drīzāk apmierina	Pilnībā apmierina

5.8. Ko jūs mainītu attiecībā uz ēdienkartēm vai ēdieniem, kas tiek pasniegti šajā aprūpes centrā, ja varētu?

____

6. faktors - Aprūpes kvalitāte

6.1. Vai jums šķiet, ka šajā aprūpes centrā saņemat kvalitatīvu aprūpi un citus pakalpojumus?

Nepiekrītu	Drīzāk nepiekrītu	Neitrāli (ne piekrītu, ne nepiekrītu)	Drīzāk piekrītu	Piekrītu

6.2. Vai jūs domājat, ka aprūpētājiem patīk viņu darbs?

Nepiekrītu (visiem vai gandrīz visiem nepatīk)	Drīzāk nepiekrītu (lielai daļai nepatīk)	Neitrāli (apmēram pusei nepatīk, pusei patīk)	Drīzāk piekrītu (lielai daļai patīk)	Piekrītu (visiem vai gandrīz visiem patīk)

6.3. Vai jūs domājat, ka sociālā darba speciālistiem (sociālais darbinieks, sociālais rehabilitētājs, sociālais aprūpētājs) un medmāsām patīk viņu darbs?

Nepiekrītu (visiem vai gandrīz visiem nepatīk)	Drīzāk nepiekrītu (lielai daļai nepatīk)	Neitrāli (apmēram pusei nepatīk, pusei patīk)	Drīzāk piekrītu (lielai daļai patīk)	Piekrītu (visiem vai gandrīz visiem patīk)

6.4. Vai jums šķiet, ka darbinieki patiesi cenšas nodrošināt aprūpi un pakalpojumus, kas atbilst jūsu personīgajām vēlmēm? Vai darbiniekam darot savus darbus , ņem vērā klienta viedokli? Piemēram istabu iztīra plkst. 9.00, bet jūs vēlaties ka to izdara plkst. 10.00

Nepiekrītu	Drīzāk nepiekrītu	Neitrāli (ne piekrītu, ne nepiekrītu)	Drīzāk piekrītu	Piekrītu

6.5. Vai jūs zināt, kas ir jūsu ārsts (t.i., aprūpes centra atbildīgais medicīniskais darbinieks, personīgais ārsts ārpus aprūpes centra)?

Noteikti zinu	Neesmu pārliecināts par ārstu vai atbildīgo medicīnas darbinieku)	Nezinu ne ārstu, ne atbildīgo medicīnas darbinieku

6.6. Vai jūs domājat, ka ģimenes ārsts uzklausa jūsu bažas un iekļauj jūsu vēlmes centra nodrošinātajā aprūpē?

Nekad	Reti	Dažreiz	bieži	Vienmēr

6.7. Vai jūs domājat, ka SAC atbildīgais medicīnas darbinieks uzklausa jūsu bažas un iekļauj jūsu vēlmes centra nodrošinātajā aprūpē?

Nekad	Reti	Dažreiz	bieži	Vienmēr

6.8. Vai jums liekas, ka darbinieki ir patiess attiecībās ar jums?

Nekad	Reti	Dažreiz	bieži	Vienmēr

6.9. Vai jūs uzticaties darbiniekiem, kad viņi jums kaut ko saka?

Nekad	Reti	Dažreiz	bieži	Vienmēr

6.10. Vai jums šķiet, kas (fizioterapeits, ergoterapeits) jūsos ieklausās un jums palīdz nodarbību laikā?

Nekad	Reti	Dažreiz	bieži	Vienmēr

6.11. Vai jums šķiet, ka fizioterapijas un ergoterapijas nodarbības tiek labi koordinētas ar aprūpes personālu un atbildīgo medicīnas darbinieki, lai pienācīgi risinātu sāpju mazināšanu?

Nekad	Reti	Dažreiz	bieži	Vienmēr

6.12. Vai jums liekas, ka jums tiek dots pietiekami daudz laika fizioterapijas un ergoterapijas nodarbības ?

Nepiekrītu	Drīzāk nepiekrītu	Neitrāli (dažreiz jā, dažreiz nē)	Drīzāk piekrītu	Piekrītu

6.13. Vai ir kādi jautājumi, kurus ir izvirzījuši klienti vai viņu tuvinieki SAC aprūpes padomē, kurus, jūsuprāt, šīs aprūpes iestādes vadītāji nav pienācīgi risinājuši?

Nekad nerisina	Drīzāk nerisina	Neitrāli (dažreiz risina, dažreiz nē)	Drīzāk risina	Vienmēr risina

---

Kuri no faktoriem, par kuriem tikko runājām, **jums liekas vissvarīgākie**, lai nodrošinātu dzīves kvalitāti sociālās aprūpes centrā. Sakārtojiet tos prioritārā secībā, kur 1.vietā ir jums vissvarīgākais, kas visvairāk ietekmē dzīves kvalitāti, bet 6.vietā ir vismazāk svarīgais.

- **1. Patstāvība un cieņpilna attiekme -** darbinieku cieņpilna attieksme un individuāla pieeja**.**
- **2. Attiecības** ar darbiniekiem un citiem klientiem **–** attiecību kvalitāte un pozitīva sadarbība.
- **3. Aktivitātes** – interesantas, daudzveidīgas un saturīgas aktivitātes gan iekštelpās, gan ārpus SAC.
- **4.** Komfortabla un vizuāli pievilcīga **vide.**
- **5. Ēdiens –** pārtikas kvalitāte, daudzums, daudzveidība**.**
- **6. Aprūpes kvalitāte** – darbinieki, kas labi izpilda savu darbu un ņem vērā klienta vēlmes.
