# Supplementary figures and images for: Enhancing Quality of Life: Key Factors from Long-Term Social Care Residents’ Perspectives
Source: Int J Environ Res Public Health. 2025 Feb 6;22(2):231. doi: 10.3390/ijerph22020231 (PMC11855746; doi:10.3390/ijerph22020231)

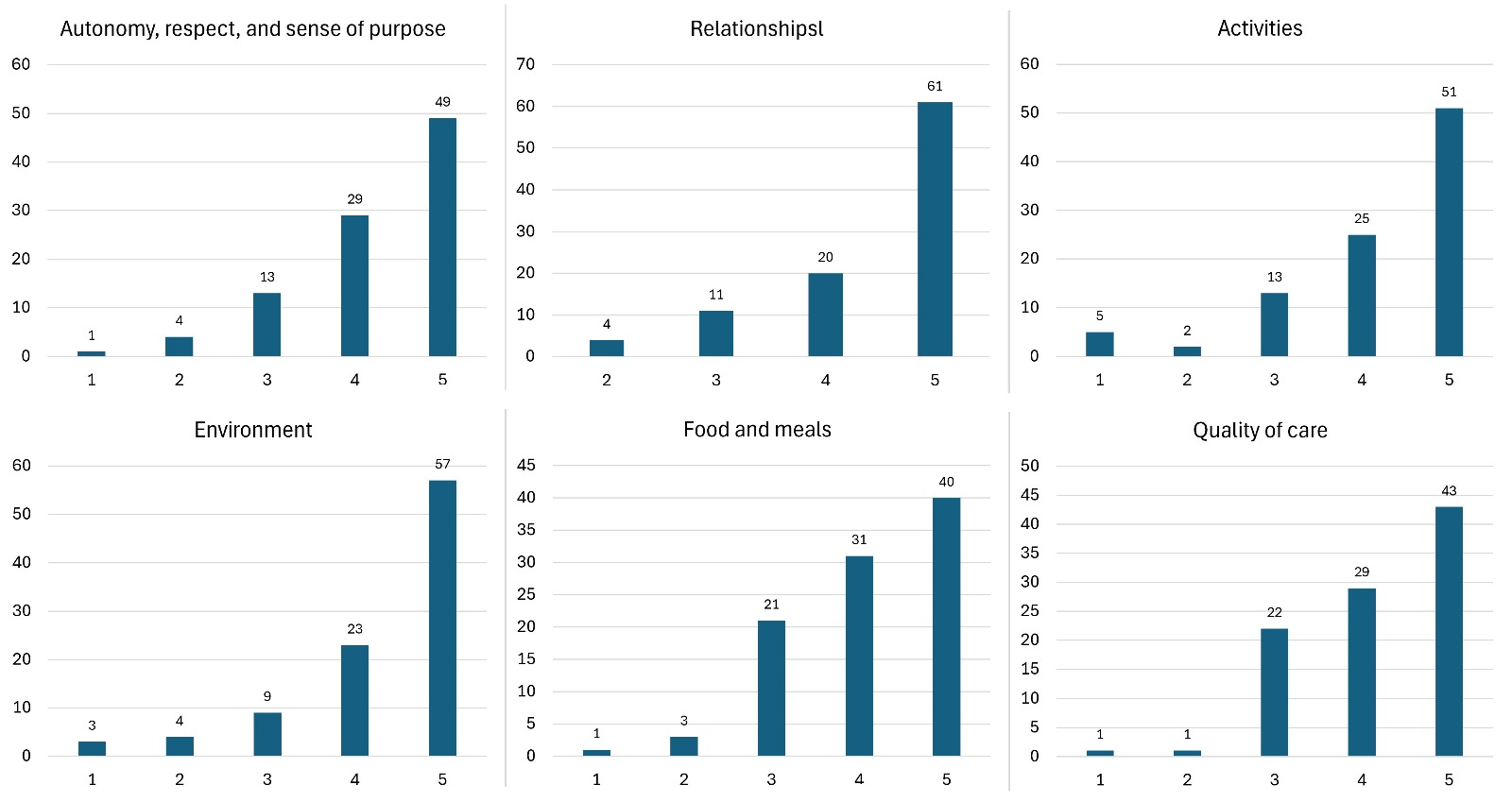

Supplement: Supplementary file 1 [file ijerph-22-00231-s001.zip › ijerph-3300022-Distribution of quality-of-life scores depicted as bar chart figures.png]
